# Impact of Zinc Deficiency on the Severity of Pneumonia in Pediatric Patients: A Cross-Sectional Study

**DOI:** 10.7759/cureus.98383

**Published:** 2025-12-03

**Authors:** Makvala Natroshvili, Maia Chkhaidze

**Affiliations:** 1 Pediatrics, New Vision University, Tbilisi, GEO; 2 Pediatrics, I. Tsitsishvili Children’s Clinic, Tbilisi, GEO; 3 Pediatrics, Caucasus International University, Tbilisi, GEO; 4 Pediatrics, Georgian Academy of Pediatrics, Tbilisi, GEO

**Keywords:** crp (c-reactive protein), micronutrients and immunity, pediatric pneumonia, pneumonia severity assessment, serum zinc levels, zinc deficiency

## Abstract

Pneumonia remains one of the leading causes of morbidity and mortality in children worldwide, particularly in low- and middle-income countries. Despite advances in vaccination, antimicrobial therapy, and supportive care, severe pneumonia continues to pose a major public health challenge. Identifying modifiable risk factors that influence disease severity is essential for developing more effective preventive and therapeutic strategies.

Zinc is a vital trace element involved in numerous physiological processes, including immune response regulation, maintenance of epithelial barrier integrity, and antioxidant defense. Zinc deficiency impairs both innate and adaptive immune functions, thereby increasing susceptibility to infections, especially respiratory infections in children.

The aim of this study was to describe serum zinc levels in children hospitalized with community-acquired pneumonia and to assess whether an association exists between zinc levels and clinical severity. A total of 44 patients were included in the analysis. The results showed that children with more severe pneumonia tended to have lower serum zinc levels, and these findings indicate an association rather than a causal relationship.

These results suggest a potential link between zinc status and pneumonia severity; however, due to the cross-sectional design and the presence of unmeasured confounding factors, no causal interpretation can be made. Further large-scale, prospective studies are needed to clarify this relationship and to determine the clinical implications of zinc status in pediatric respiratory infections.

## Introduction

Pneumonia is an inflammation of the lower respiratory tract and alveoli, caused by the invasion of infectious agents into the lungs, and is characterized by respiratory symptoms, fever, and radiographic evidence of pulmonary infiltrates [1-2]. Community-acquired pneumonia (CAP) refers to pneumonia that develops in otherwise healthy children as a result of infections acquired outside of the hospital setting. In high-income countries, pneumonia is a leading cause of morbidity, while in low- and middle-income countries (LMICs), it remains a major cause of both morbidity and mortality. In resource-abundant countries, the annual incidence of pneumonia is estimated to be 3.3 per 1000 in children younger than five years and 1.45 per 1000 in children 0 to 16 years [1]. Moreover, approximately half of children under five diagnosed with pneumonia require hospitalization. In a systematic review, the annual incidence of pneumonia in children younger than five years from resource-limited countries in 2015 was estimated to be 231 per 1000; 50% to 80% of children with severe pneumonia required hospitalization [3].

Zinc plays a critical role in the development and function of the immune system [4]. It is involved in tissue growth and repair, wound healing, and the synthesis of testicular hormones. Zinc intake is closely associated with dietary protein consumption; thus, zinc deficiency commonly arises in conditions of inadequate nutrition. Clinical manifestations of severe zinc deficiency include growth retardation, diarrhea, primary hypogonadism, dermatological disorders, altered taste and smell, as well as impaired immunity and reduced resistance to infections. Zinc deficiency also contributes to an increased frequency of acute illnesses in pediatric populations. Insufficient zinc intake represents a major global health issue, particularly in developing countries. Approximately 17.3% of the global population is considered at risk of zinc deficiency due to inadequate intake, which in turn increases the risk of diarrhea and pneumonia by nearly 20% [5,6]. Zinc deficiency is recognized as one of the most widespread micronutrient deficiencies [7] and is associated with high morbidity in LMICs [8].

The recommended daily intake of zinc ranges from 2 to 9-11 mg/day, depending on age [9]. The primary dietary sources of zinc include animal products (meat, fish), as well as certain plant-based foods (cashews, pumpkin seeds, chickpeas), and fortified cereals.

Several studies have demonstrated that zinc supplementation reduces the prevalence of zinc deficiency, improves weight gain in undernourished children (mean increase of 0.43 kg) [10], and decreases both the frequency of diarrheal diseases and diarrhea-related mortality [11,12]. Furthermore, zinc supplementation has been shown to shorten the duration of respiratory symptoms such as the common cold [13] and to reduce the incidence of radiologically confirmed pneumonia by an average of 20% [14-15]. Taken together, these findings underscore the high risk of zinc deficiency in the pediatric population.

Therefore, the present study aims to evaluate serum zinc levels in pediatric patients hospitalized with CAP and to investigate the potential association between zinc deficiency and the severity of the disease. By identifying whether low serum zinc concentrations correlate with more severe clinical manifestations and inflammatory responses, this research seeks to highlight the role of zinc as a modifiable factor influencing pneumonia outcomes in children.

## Materials and methods

The study was conducted in Georgia at the I. Tsitsishvili Children's Clinic in Tbilisi, in both the pediatric and intensive care units. A cross-sectional design was employed between June 1, 2024, and February 1, 2025. A total of 44 patients aged 1-18 years with clinical signs of pneumonia were enrolled. The diagnosis of pneumonia was confirmed radiologically using a chest X-ray.

Sampling technique and study population

A consecutive sampling method was employed, whereby all eligible patients admitted during the study period who met the inclusion criteria were invited to participate. A total of 44 patients were enrolled after obtaining informed consent from parents or legal guardians.

The study included children aged 1 to 18 years who demonstrated both clinical symptoms and radiographic confirmation of pneumonia on chest X-ray. Only patients with CAP diagnosed at the time of admission were enrolled. Children were excluded if they were preterm neonates, younger than one year of age, or had chronic comorbid conditions such as congenital heart disease, cystic fibrosis, bronchiectasis, or primary or secondary immunodeficiency. In addition, non-immunized children, those diagnosed with hospital-acquired pneumonia, and those with any condition that could potentially worsen the course of pneumonia - such as malnutrition, chronic anemia, or congenital lung malformations - were excluded from the study.

Potential confounding factors, such as nutritional status, prior antibiotic use, immunization history, and presence of underlying chronic conditions, were identified and minimized through strict inclusion/exclusion criteria and consistent diagnostic evaluation across all participants. Nutritional status was assessed only through clinical screening. During the study process, it was not possible to measure serum albumin or other micronutrient levels.

Serum zinc concentration was measured in all participants. The analysis was performed in a fasting state (8-12 hours had passed since the last meal). The sample was centrifuged within a maximum of 30 minutes after collection, the separated plasma was transferred to a test tube within a maximum of four hours, and sent to the Limbach Laboratory Center, observing the temperature regime. Serum zinc deficiency was defined as <60 μg/dL (9.2 μmol/L). The reference interval was applied by the Limbach Laboratory Center. The severity of pneumonia was assessed using radiological and laboratory findings (complete blood count and C-reactive protein (CRP) testing) and following the criteria outlined in Table 1, which was developed by the authors based on the National Clinical Practice Guidelines.

**Table 1:** Classification of pneumonia severity based on the National Clinical Practice Guideline for the Management of Community-Acquired Pneumonia in Children (Ministry of Health of Georgia, 2015), with minor adaptations for research standardization. Severity criteria were derived from the national guideline but adapted slightly for research purposes. Overlapping clinical descriptors were consolidated into broader categories (moderate/severe), age-specific thresholds for tachypnea and hypoxemia were standardized, and outpatient-specific elements were excluded, as they were not applicable to hospitalized patients. SaO_2_: arterial oxygen saturation

Mild pneumonia	Moderate/severe pneumonia
Temperature <38.5 °C	Temperature >38.5 °C
Tachypnea but <70/min in infants, <50/min in older children	Respiratory rate >70/min in infants, >50/min in older children
Mild or no chest retraction	Moderate to marked chest retraction
Mild breathing difficulty	Significant breathing difficulty
No nasal flaring	Nasal flaring
Mild breathing difficulty	Apnea
Normal skin color	Cyanosis
Normal mental status	Altered mental status (agitation/lethargy)
SaO_2_ ≥ 92% on room air	SaO_2_ <92% on room air
Feeding well (infants), no vomiting or dehydration	Feeding difficulties (infants) or dehydration (older children)
Normal heart rate	Tachycardia
Capillary refill <2 sec	Capillary refill >2 sec

The study protocol was reviewed and approved by the Institutional Review Board (IRB) of I. Tsitsishvili Children’s Clinic, Tbilisi, Georgia (Approval No. 4, dated May 16, 2024). All research procedures were conducted in accordance with the ethical standards of the institutional and national research committees and with the principles of the 1964 Declaration of Helsinki and its subsequent amendments. Written informed consent was obtained from all participants’ parents or legal guardians prior to their inclusion in the study.

Statistical analyses were performed using IBM SPSS Statistics for Windows, Version 26 (Released 2018; IBM Corp., Armonk, New York, United States). Data normality was assessed using the Shapiro-Wilk test. Associations between serum zinc levels and clinical parameters (CRP, oxygen saturation (SpO_2_), respiratory rate (RR), and pneumonia severity) were evaluated using Pearson’s correlation coefficient. One-way analysis of variance (ANOVA) was employed to compare zinc levels across severity subgroups. A p-value <0.05 was considered statistically significant.

## Results

A total of 44 children who met all predefined inclusion criteria were enrolled in the study. Since patients were included directly at the time of admission based on strict eligibility criteria, the number of screened or excluded children was not recorded as a separate dataset. Only those who fulfilled the criteria for CAP and had no signs of chronic illness, malnutrition, or incomplete immunization were included.

The age of participants ranged from 1 to 18 years, with the majority belonging to the younger age groups; however, detailed age-stratified analysis was not performed at this stage of the study. Nutritional status was assessed clinically during screening, and no child with clinical signs of malnutrition or growth retardation was enrolled. Immunization status was evaluated based on caregiver report and medical documentation, and children with incomplete routine immunization (excluding seasonal influenza vaccination) were not included.

Descriptive statistical analysis revealed that the mean serum zinc concentration was 9.65 ± 2.42 μmol/L (range: 4.5-14.5 μmol/L). The mean RR was 38.41 ± 7.71 breaths per minute. The mean value of the inflammatory marker CRP was 105.04 ± 104.68 mg/L (range: 3.10-472.69). The mean SpO_2_ was 91.90 ± 6.04% (range: 65-99), and the mean percentage of granulocytes was 74.20 ± 13.06% (range: 24-94). These findings indicate that the study cohort included children with both relatively mild and severe clinical conditions (Table 2).

**Table 2:** Descriptive statistics of key variables summarize the mean, minimum, and maximum values for serum zinc levels, respiratory rate, CRP, oxygen saturation, and granulocyte percentage. This table provides an overview of the general clinical and laboratory characteristics of the study population and reflects findings derived from the complete dataset of all 44 patients included in the study. CRP: C-reactive protein; SpO_2_: oxygen saturation

Variable	Mean	Min	Max	SD
Serum zinc (μmol/L)	9.65	4.5	14.5	2.43
Respiratory rate (breaths/min)	38.41	26	56	7.71
CRP (mg/L)	105.04	3.1	472.7	104.7
SpO_2_ (%)	91.9	85	95	2.58
Granulocytes (%)	74.23	55.1	88.8	10.19

Normal distribution of serum zinc levels was confirmed by the Shapiro-Wilk test (p = 0.583), as presented in Table 3.

**Table 3:** Test of normality * This is a lower bound of the true significance; ^a^ Lilliefors significance correction.

Variable	Kolmogorov-Smirnov^a^			Shapiro-Wilk		
	Statistic	df	Sig.	Statistic	df	Sig.
Zn_serum	0.075	44	0.200*	0.979	44	0.583

Correlation analysis demonstrated that zinc concentration was significantly and negatively associated with the inflammatory marker CRP (r = -0.509, p < 0.001), RR (r = -0.363, p = 0.016), and pneumonia severity category (r = -0.395, p = 0.008). A trend toward a positive association was observed between zinc levels and SpO_2_ (r = 0.289, p = 0.057). These results indicate that zinc deficiency is associated with elevated CRP, granulocytosis, and greater severity of pneumonia (Table 4).

**Table 4:** Correlations between serum zinc and clinical-laboratory parameters further details the strength and direction of these associations, indicating that lower zinc levels correlate with elevated CRP, higher granulocyte count, and more severe pneumonia. CRP: C-reactive protein; SpO_2_: oxygen saturation

Variable	Pearson r (p)
Respiratory rate (RR)	-0.363 (p = 0.016)
SpO_2_ (%)	0.289 (p = 0.057)
WBC	-0.149 (p = 0.336)
Granulocytes (%)	-0.402 (p = 0.007)
CRP (mg/L)	-0.509 (p < 0.001)
Pneumonia severity category	-0.395 (p = 0.008)

One-way ANOVA revealed that serum zinc levels differed significantly across pneumonia severity groups (F = 3.286, p = 0.030). Specifically, the mean zinc concentration in patients with unilateral pneumonia (right- or left-sided) was 10.53 μmol/L, whereas in the severe categories - pneumonia associated with pleuritis or respiratory failure - zinc levels were significantly lower (7.8-8.6 μmol/L). This trend indicates that as the disease progresses, serum zinc levels decrease consistently.

## Discussion

The findings of this study suggest that lower serum zinc levels are associated with clinical severity and increased inflammatory response in children hospitalized with CAP. Although the associations observed between zinc concentrations, CRP levels, hypoxemia, and tachypnea align with previously published evidence on the immunomodulatory role of zinc, the results should be interpreted with appropriate caution. Given the cross-sectional design, the study can identify correlations but cannot determine whether zinc deficiency contributes to the development of severe pneumonia or whether lower zinc levels arise secondarily as part of the acute-phase response.

Our findings are consistent with previous research indicating that zinc plays a key role in immune regulation and inflammatory control during respiratory infections. Several clinical trials conducted in LMICs have also reported that zinc supplementation may reduce the duration or severity of pneumonia episodes in children [16,17]. While these external data support the biological plausibility of an association between zinc deficiency and more severe disease, the present study was not designed to evaluate causality or the therapeutic effects of zinc supplementation. Instead, it contributes additional observational evidence to a growing body of literature suggesting that zinc status may be relevant in pediatric respiratory illnesses across various socioeconomic settings - not only in LMICs but also in high-income countries, where selective eating habits, restricted diets, or chronic conditions can also predispose children to zinc deficiency.

From a public health standpoint, the observed association underscores the importance of clinically assessing nutritional status in children presenting with respiratory infections. Although Georgia does not implement mandatory zinc fortification programs, micronutrient insufficiencies remain a recognized concern in several pediatric subgroups. Therefore, monitoring zinc levels may be informative when evaluating children with moderate or severe pneumonia. Nevertheless, any potential clinical implications must be viewed as preliminary, and further prospective and interventional studies are necessary to clarify whether improving zinc status could influence the clinical course or outcomes of pneumonia.

This study has several limitations that should be acknowledged. First, the cross-sectional design precludes the determination of temporal or causal relationships. Serum zinc is a negative acute-phase reactant, and concentrations may decrease during systemic inflammation, independent of true nutritional deficiency. Although strict inclusion criteria were applied - excluding children with malnutrition, incomplete immunization, chronic comorbidities, or growth retardation - several other confounding factors that influence zinc levels were not assessed. These include serum albumin concentration, fasting status, dietary intake, the timing of blood sampling, and the stage of illness at the time of measurement.

Second, while pre-analytical procedures and blood collection techniques were standardized, the study was unable to measure additional biomarkers (e.g., albumin, other micronutrients, interleukin-6), which could have strengthened the interpretation of zinc-related immune response dynamics. Microbiological confirmation was also not routinely available for all patients, as culture-based diagnostics are not systematically performed in all hospitalized children with pneumonia and may not always reflect the causative pathogen.

Third, the sample size was relatively small (n=44), which limits the generalizability of the findings and reduces the statistical power to explore age-stratified or etiological subgroups. The age distribution and pneumonia severity by age groups or sex were not analyzed in detail and will require further investigation in future studies with larger cohorts.

Despite these limitations, the study provides observational evidence of an association between lower serum zinc levels and more severe clinical presentation in pediatric pneumonia. Future research using larger, prospective designs with comprehensive nutritional and immunological profiling is needed to better define these relationships and assess their potential clinical significance.

## Conclusions

To summarize, this study found that children hospitalized with CAP who presented with more severe clinical and inflammatory manifestations had lower serum zinc levels compared with those with milder disease. While these results suggest a possible association between zinc status and pneumonia severity, the cross-sectional study design does not allow conclusions regarding causality. Serum zinc concentrations may be influenced by multiple factors, including acute-phase reactions, nutritional status, and sample collection conditions, which were only partially controlled in this study. Despite these limitations, the observed associations align with previous research highlighting the potential relevance of zinc in immune function and respiratory illnesses. The findings emphasize the need for further prospective, well-controlled studies that include comprehensive nutritional assessment and additional inflammatory biomarkers to better clarify the relationship between zinc levels and pneumonia severity. Larger interventional studies are also warranted to determine whether improving zinc status could contribute to better clinical outcomes in pediatric pneumonia. Overall, the present results add to the growing body of evidence suggesting that zinc may play an important role in the clinical presentation of respiratory infections in children, but additional research is required before any causal or therapeutic conclusions can be drawn.
